# Special Issue “Bone Ontogeny, Embryology, and Homeostasis”

**DOI:** 10.3390/ijms23137212

**Published:** 2022-06-29

**Authors:** John Kelly Smith

**Affiliations:** Departments of Medical Education and Biomedical Sciences, James H Quillen College of Medicine, East Tennessee State University, P.O. Box 70300, Johnson City, TN 37614, USA; smithj@etsu.edu

The intention of this Special Issue is to provide the reader with an in-depth understanding of the ontogeny, embryology, and homeostasis of bone, with an emphasis on recent research that has contributed to our understanding of the skeletal system at the molecular level. This understanding is an essential step in our continued efforts to diagnose and treat metabolic, inflammatory, and malignant diseases of bone as they occur on Earth, and, in the not so distant future, as they occur in the microgravity of space [1]. An area of interest to the Editor which has not yet been covered in this Special Issue is the effect of physical exercise on bone homeostasis and on bone and cartilage regeneration therapies [2,3].

In this Special Issue, Kim and associates provided cutting-edge research on the potential of the intravenous administration of parathyroid-hormone-related protein minicircle DNA (mcPTHrP) vectors to enhance bone formation in osteoporosis. Using ovariectomized osteoporotic mice, the researchers showed enhanced trabecular bone structure quality, increased bone formation, and decreased bone resorption upon the administration of mcPTHrP as compared to controls, demonstrating the potential usefulness of this therapy in clinical trials [4].

Luca Ambrosio and associates provided the reader with a comprehensive review of biomaterials that are designed to selectively inhibit bone cancer progression while simultaneously reducing the loss of bone structural properties secondary to local tissue invasion. Included in this review is a discussion on recent advancements in nanocarrier-based drug delivery systems, as well as an update on multifunctional biomaterials that show promise in the treatment of bone cancer [5].

Neag and associates discussed the key role that angiotrophism between bone marrow endothelial cells and osteoblasts plays in bone development and homeostasis. They noted that specialized “type H” capillary endothelial cells help to regulate osteoblast differentiation, maturation, and migration, and discussed the intercellular cross talk between endothelial cells and osteocytes which is essential for bone formation, repair, and maintenance. They noted that recent revolutions in high-resolution imaging methodologies for bone, single-cell, and RNA sequencing technologies have enabled the identification of signaling pathways and molecular interactions that underpin these regulatory relationships, laying the foundation for the identification of new drug targets for diseases of bone formation and remodeling [6].

He and associates conducted a comprehensive literature search to investigate the possibility that extracellular vesicles (EVs) contribute to (and serve as a biomarker for) the development of osteoporosis in persons undergoing prolonged psychosocial stress. They concluded that although EVs have been shown to provide communication signals between cells in vitro, there is yet insufficient evidence to support a role of EVs in the development of osteoporosis. Their article introduces a subject that may provide fertile ground for future research [7].

Additionally, Villa-Suárez and associates provided a comprehensive review of hypophosphatasia (HPP), a rare genetic disease characterized by a decrease in the activity of tissue non-specific alkaline phosphatase. They noted that the severity of HPP is inversely related to the age at diagnosis, noting that affected infants may have short limbs, abnormally shaped chests, and soft skulls, whereas adults may lose their teeth prematurely and develop inflammatory joint disease. Their review raises the prospect of molecular intervention in this rare and poorly understood disorder [8].

These contributions to this Special Issue provide valuable insights into the diagnosis and treatment of bone disease. The Editor looks forward to future contributions to this exciting area of research.
